# Early Screening of Children With Autism Spectrum Disorder Based on Electroencephalogram Signal Feature Selection With L1-Norm Regularization

**DOI:** 10.3389/fnhum.2021.656578

**Published:** 2021-06-22

**Authors:** Shixin Peng, Ruyi Xu, Xin Yi, Xin Hu, Lili Liu, Leyuan Liu

**Affiliations:** ^1^National Engineering Laboratory for Education Big Data, Faculty of Artificial Intelligence Education, Central China Normal University, Wuhan, China; ^2^National Engineering Research Center for E-Learning, Faculty of Artificial Intelligence Education, Central China Normal University, Wuhan, China

**Keywords:** early screening, autism spectrum disorder, electroencephalogram signal, feature selection, event-related potential

## Abstract

Early screening is vital and helpful for implementing intensive intervention and rehabilitation therapy for children with autism spectrum disorder (ASD). Research has shown that electroencephalogram (EEG) signals can reflect abnormal brain function of children with ASD, and screening with EEG signals has the characteristics of good real-time performance and high sensitivity. However, the existing EEG screening algorithms mostly focus on the data analysis in the resting state, and the extracted EEG features have some disadvantages such as weak representation capacity and information redundancy. In this study, we utilized the event-related potential (ERP) technique to acquire the EEG data of the subjects under positive and negative emotional stimulation and proposed an EEG Feature Selection Algorithm based on L1-norm regularization to perform screening of autism. The proposed EEG Feature Selection Algorithm includes the following steps: (1) extracting 20 EEG features from the raw data, (2) classification with support vector machine, (3) selecting appropriate EEG feature with L1-norm regularization according to the classification performance. The experimental results show that the accuracy for screening of children with ASD can reach 93.8% and 87.5% under positive and negative emotional stimulation and the proposed algorithm can effectively eliminate redundant features and improve screening accuracy.

## 1. Introduction

Autism spectrum disorder (ASD) is a lifelong neurodevelopmental disorder involving deficits in interpersonal communication and social interactions, as well as restricted, repetitive mannerisms and interests (American Psychiatric Association, 2013). The study by Bickel et al. (2015) and Polyak et al. (2015) suggested that ASD often includes some other comorbidities, such as epilepsy, attention deficit hyperactivity disorder (ADHD), dysthymia, sleep disorders, psychiatric disorders, and gastrointestinal disorders. At present, autism has become a kind of disease with the highest rate of disability in children, which seriously endangers the physical and mental health of patients. Barton et al. (2012) stated that early screening and early intensive intervention can effectively improve the social interaction and cognitive development of children with ASD. Therefore, it is of vital realistic significance to explore the early screening methods for children with ASD.

In recent years, the screening methods of autism based on electroencephalogram (EEG) have drawn more attention. EEG can be used for real-time tracking of the neural activity of the brain in milliseconds and detecting subtle differences in neural oscillations more sensitively (Chen et al., 2021), thus quickly and accurately distinguishing children with ASD and typically developing (TD) children with EEG signals. Shams and Wahab (2013) collected EEG data among children with ASD and TD children in the resting state of eyes open and closed, extracted time-domain features of these EEG data, and then classified them using a neural network with multi-layer perception (MLP) technology. Esguerra et al. (2012) conducted a non-linear analysis of EEG in children with ASD and explored the differences among TD children, children with moderate ASD, and children with deep ASD. Tierney et al. (2012) collected EEG signals in the resting state of children with ASD and TD children and performed the power spectrum analysis. The experimental results obtained in the study by Tierney et al. (2012) showed that the power spectrum of high-risk infants was lower than that of low-risk infants. Acharya et al. (2015) utilized multiple entropy methods to identify normal and interstitial EEG signals and showed that spectral entropy, permutation entropy (PE), and sample entropy are high-resolution features to distinguish normal and epileptic EEG signals. Zhao et al. (2019) collected EEG signals of 37 children with ASD and 38 TD children in resting state for 5 min, and they computed EEG features such as approximate entropy, sample entropy, wavelet entropy, and sequencing entropy. The feature selection method suggested by Zhao et al. (2019) combined the permutation test with a support vector machine (SVM) classifier, and the highest screening accuracy rate of children with ASD was 84.55%. Dong et al. (2021) developed a subject sensitive EEG discrimination for ASD evaluation with fast reconstructable CNN driven by reinforcement learning. Lei et al. (2016) investigated the brain activity characteristics of children with ASD during virtual driving environment by analyzing EEG signals from the perspective of neuroergonomics. The method of the shift average sample entropy is proposed to deal with EEG signals in the resting and the virtual driving environments. The results in the study by Lei et al. (2016) showed that the sliding mean sample entropy of children with ASD was generally lower than that of TD children, especially in the prefrontal, temporal, parietal, and occipital functional areas. Fan et al. (2017) developed the group-level classification models capable of recognizing affective states and mental workload of individuals with ASD during driving skill training. The offline EEG-based group-level classification models are feasible for recognizing binary low and high intensity of affect and workload of individuals with ASD in the context of driving. However, the applicability of the models in an online adaptive driving task requires further development.

Given a brain stimulus, such as light, pictures, music, and emotion, dynamic EEG signals of the brain can be collected under these stimulations, which is called event-related potential (ERP). ERP is considered as one of the most effective methods to monitor cognitive processes. Relevant researchers believed that ERP can be used to screen various pathological nerve disorders and achieve higher screening accuracy, such as ADHD and ASD (Castro-Cabrera et al., 2010). Meanwhile, some studies have shown that individuals with ASD have a poor perception of facial expression of others (Kiyoto et al., 2007). However, fewer studies focus on the difference of perception of facial emotion between children with ASD and TD children with ERP methods. Furthermore, the experimental design of ERP for facial emotional stimuli is also scanty in the existing literature. The randomness and non-stationarity of EEG signal also make autism screening based on ERP more complicated. Therefore, it is of vital significance to construct a screening method for children with ASD based on EEG signals.

In general, whether screening methods for children with ASD based on EEG signals can work effectively involves several key factors such as suitable EEG characteristic parameters, high robustness of the recognition algorithm, and low computation complexity of recognition algorithm. Feature selection in EEG signals is the key to the performance of screening algorithms for children with ASD. In the existing work, however, only a few kinds of features were examined, and why those robust features contribute to cross-subject emotion recognition was not studied. Therefore, the extracted features are not enough to express the differentiated information in the EEG signal, and the complementarity among features is weak. On the other hand, in the EEG candidate feature set, the removal of irrelevant redundant features can greatly improve the classification performance. At present, the two typical automatic feature selection techniques are the filter-based strategy and the wrapper-based strategy (Guyon and Elisseeff, 2003). Most studies on autism screening based on EEG signals adopt the filter-based feature selection method. However, the filter-based method fails to consider the complementarity between various EEG features and cannot effectively remove the redundant EEG features. When compared with the filter-based method, the wrapper-based method considers the interaction between EEG features and can search the most suitable feature subset more effectively, with higher accuracy and stronger generalization ability.

In this study, we proposed a linear SVM with L1-norm feature selection for screening children with ASD under positive and negative emotional stimuli. First, the experimental paradigm of ERP was constructed to stimulate the positive and negative emotions of the subjects and to extract evoked EEG signals. Then, we established the mapping relationship between EEG signal and social and emotional behaviors and analyzed the differences between TD children and children with ASD in social affective cognition. On the other hand, in the existing work, only a few kinds of EEG signal characteristics have been studied, and the representation ability of extracted features is weak for screening children with ASD. This study extracts a wider range of 20 types of EEG features for utilization in autism screening and analyzes their effectiveness in distinguishing children with ASD and TD children under positive and negative emotional stimuli. Taking account of the redundancy and complementarity of multiple features, the linear SVM of L1-norm feature selection algorithm was employed in our study to improve the screening accuracy of children with ASD. Provided that utilizing the conventional linear SVM classification, the highest screening accuracy for children with ASD was 81% and 78% under positive and negative emotional stimuli, respectively. Our experiment shows that screening accuracy for children with ASD can reach 93.75% and 87.5% under the positive and negative emotional stimuli using the L1-norm feature selection algorithm. The above-mentioned results verify that the L1-norm can remove redundant features and select better characteristics, which improve screening accuracy for children with ASD.

## 2. Methodology

### 2.1. Participants

A total of 80 children were recruited in this study. Written informed consent from parents was obtained prior to participation. The participants were divided into ASD group and TD group. According to the experimental cooperation of children recorded by the experimenters, children with high coordination degree were selected as follows: The ASD group consisted of 32 children aged 4–6 years (*M* = 4.98 years, SD = 14 months), and the TD group consisted of 32 children (*M* = 5.2 years, SD = 8 months).

Children with ASD were recruited from a special school. The inclusion criteria for the ASD group included (1) diagnosis in accordance with DSM-V (American Psychiatric Association, 2013; Lobar, 2016), (2) double-blind diagnosis by two directors or deputy directors of developmental-behavioral pediatrics, (3) aged 2–7 years, (4) no severe respiratory diseases, schizophrenia, epilepsy, and other brain organic diseases, and (5) normal development of the visual system.

The typically developing children were recruited from standard kindergartens. The inclusion criteria for the TD group included (1) matched age with the ASD group, (2) no suspected or diagnosed psychiatric disorder and/or other developmental delay or learning disabilities, and (3) normal development of the visual system.

### 2.2. Device

**Figure 1:**
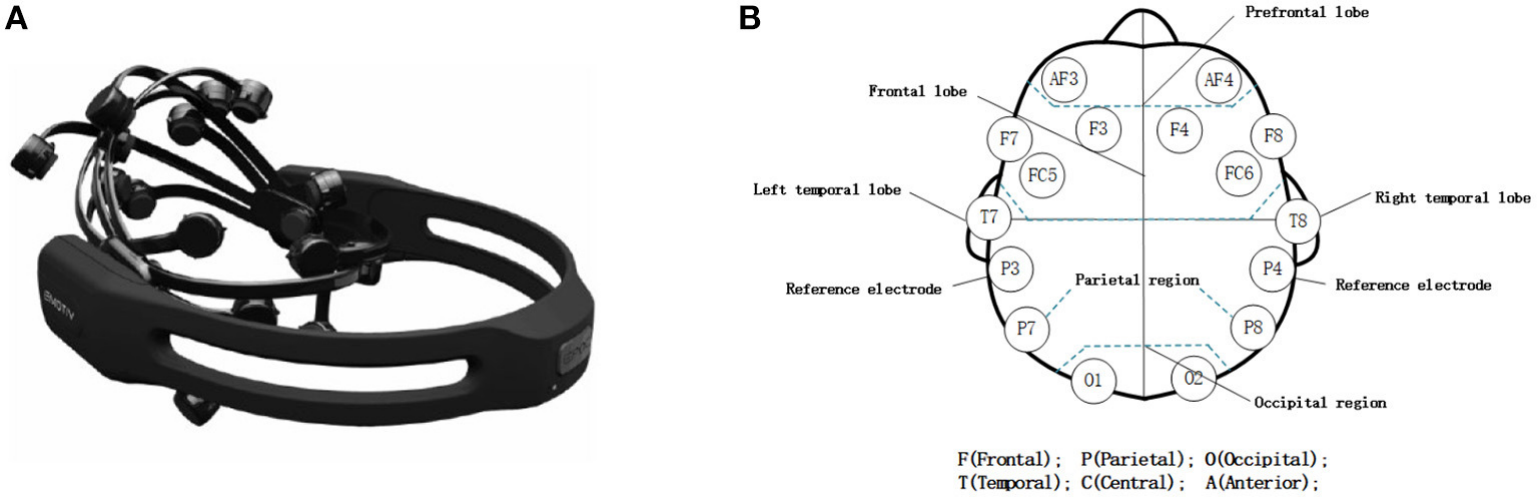
**(A)** The device EMOTIV EPOC+ and **(B)** the locations of its electrodes.

In this study, EMOTIV EPOC+ (Emotiv Inc., San Francisco, USA) (Badcock et al., 2013, 2015; Duvinage et al., 2013) was used to record EEG signals of the participants, as shown in Figure 1A. The EMOTIV EPOC+ is a portable high-resolution EEG system with 14 data acquisition electrodes (i.e., AF3, F7, F3, FC5, T7, P7, O1, O2, P8, T8, FC6, F4, F8, and AF4) and 2 reference electrodes (P3 and P4). As shown in Figure 1B, the locations of electrodes follow the international 10–20 system, which provides good coverage of the frontal and prefrontal lobes and the temporal, parietal, and occipital lobes. The device is easy to set up and wear, i.e., wet the sensors with saline and install them on the headset, and then wear the headset and adjust the position of the reference electrode until the electrode indicator light reaches 100%. After wearing, EEG signals can be sent to a PC or laptop over a Bluetooth wireless connection.

### 2.3. Materials

In order to elicit positive and negative emotions of children, affection-evoked movie clips were prepared by five psychologists. The movie clips were initially screened according to the following three criteria: (1) reasonable duration—the fatigue caused by watching for a long time will affect the emotional subjective experience, (2) easy to understand the meaning—the test requires to obtain the emotional information of children in two groups during a short time, and the unclear meaning of the movie clips may affect the emotional response time of participants, and (3) effective emotional elicitation—the movie clips can effectively elicit the emotions of participants, that is, positive and negative emotions (Rottenberg et al., 2002). Finally, 20 eligible movie clips (average duration: 28.9 s) were allowed to enter the stage of emotional elicitation effect evaluation.

A total of 10 volunteers were recruited to evaluate the emotional elicitation effect of each movie clip by self-report questionnaire. Our self-report questionnaire was designed referring to that presented by Gross and Levenson (1995). The questionnaire evaluated six categories of emotions elicited by movie clips, including joy, happiness, interest, sadness, disgust, and fear. Each category was scored according to the 9-point Likert scoring method, from 0 (none) to 8 (extremely intense). The higher the score, the higher the emotional intensity elicited by movie clips. The movie clips with a high score were selected and edited into AVI videos using the multimedia editing software Adobe Premiere. The video resolution was 720 × 576 pixels (i.e., 25.0 FPS). The positive emotion elicitation video consists of six movie clips (total duration: 94 s), including a baby playing with his mother or pet or some funny embarrassing incidents. The negative emotion elicitation video consists of two movie clips (total duration: 120 s) about a baby being given an injection.

### 2.4. Procedure

Empathy refers to an individual's understanding and speculation of others' inner state and behavior. The clinical manifestations of empathy deficiency in autism are impaired emotional cognitive ability and expression imitation ability. In view of the fact that children with ASD may have abnormal empathy function, the aim of our experiment was to analyze the difference in the brain activity between the TD children and children with ASD when watching positive/negative emotional elicitation materials by EEG signals. The experiment was performed independently for each child in a 102 isolation room. A concrete procedure is as follows: (1) explain the experimental requirements and procedures to parents and children, (2) help participant to wear the device EMOTIV EPOC+, (3) seat the participant in a chair 50–60 cm away from the screen and ask them to look at the star fixation point with both their eyes straight ahead, (4) play a piece of light music to make participant relaxed and then begin to record the EEG data, (5) play the positive/negative emotional elicitation materials and continue to record the EEG data. While playing the materials, the response of the participant was observed and recorded. If the participant did not cooperate, the recorded EEG data were excluded and retested, (6) After watching an emotional elicitation material, the participant could rest for a few minutes, and (7) repeat steps (1)–(6) to watch the other materials. Before the data collection, a parent guides his/her child to cooperate with the experimental procedures. In the process of data collection, he/she sits beside the child at a certain distance and monitors the behavior of the child in order to evaluate if the child feels comfortable. When the stimulus material is played, a parent stays quiet to ensure that the attention of his/her child is focused on the stimulus material. All the valid data were sent to a PC and saved in the corresponding folder.

### 2.5. Data Preprocessing

**Figure 2:**
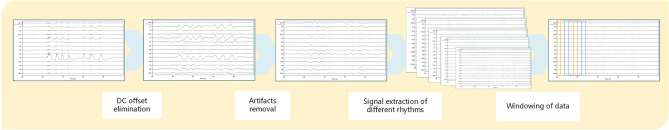
Flowchart of the data preprocessing.

To extract the effective features from the EEG data, preprocessing to the raw EEG data is necessary to eliminate the noise and trivial information. The data preprocessing includes four steps, namely, direct current (DC) offset elimination, artifacts removal, signal extraction of different rhythms, and windowing of data. The flowchart of the data preprocessing is shown in Figure 2.

(1) DC offset elimination: The method to remove the DC offset that followed the instructions of the tool EMOTIV PRO was to subtract the average value from the whole data channel, and use a 0.16-Hz first-order high-pass filter to remove the noise.
(2) Artifacts removal: In this study, the independent component analysis (ICA) was used to remove the artifacts such as electrooculogram, ECG, and electromyography (Jung et al., 1998). Being one of the current most popular blind source separation methods, ICA decomposes the observed data into several independent components and estimates mix matrix by the optimization algorithm. Each artifact was considered as one of the independent components separated from the EEG source data.
(3) Signal extraction of different rhythms: To explore the difference of the EEG in different rhythms between ASD group and TD group, a finite impulse response digital band-pass filter based on Hanning window was used to extract five rhythms of the EEG, namely, theta (4–8 Hz), alpha (8–12 Hz), low beta (12–16 Hz), high beta (16–25 Hz), and gamma (25–45 Hz).
(4) Windowing of data: After filtering, the data were split into fixed 4s windows with an overlapping size of 2s.

### 2.6. Handcrafted Feature Extraction

The EEG signal is inherently complex, non-linear, non-stationary, and random. To extract valuable information from the EEG signals, various handcrafted features are presented to describe the EEG signals. However, a single handcrafted feature cannot represent the EEG signal comprehensively. To this end, the study by Li et al. (2018) explores 18 handcrafted features including those for cross-subject emotion recognition. This study follows their work and adds two more features (i.e., sample entropy and differential entropy) which are widely used in the EEG-based emotional analysis to construct the candidate feature set, including (1) peak–peak mean, (2) mean square value, (3) variance, (4) power sum, (5) maximum power spectral frequency, (6) maximum power spectral density, (7) Hjorth parameter: activity, (8) Hjorth parameter: mobility, (9) Hjorth parameter: complexity (Hjorth, 1970), (10) correlation dimension, (11) Kolmogorov entropy (Schuster, 1995), (12) approximate entropy (Delgado-Bonal and Marshak, 2019), (13) sample entropy (Delgado-Bonal and Marshak, 2019), (14) Lyapunov exponent, (15) singular spectrum entropy, (16) permutation entropy, (17) C0 complexity, (18) Shannon entropy, (19) power spectral entropy, and (20) differential entropy (Duan et al., 2013). All the features were normalized before further analysis (Table 1).

**Table 1:** Handcrafted features used for autism spectrum disorder/typically developing (ASD/TD) classification.

**Features**	**Formulation**
1. Peak-Peak Mean (PPM)	PPM $=\frac{1}{H}\overset{H}{\underset{i=1}{\sum }}\left(x\left(P_{i}\right)-x\left(T_{i}\right)\right)$ Where, *P*_*i*_ and *T*_*i*_ are the time indexes of peak and trough, respectively.
2. Mean Square Value (MSV)	MSV $=\frac{1}{N}\overset{N-1}{\underset{n=0}{\sum }}{x\left(n\right)}^{2}$
3. Variance (VAR)	VAR $=\sigma^{2}=\frac{1}{N}\overset{N-1}{\underset{n=0}{\sum }}{\left[x\left(n\right)-\bar{x}\right]}^{2}$, $\bar{x}=\frac{1}{N}\overset{N-1}{\underset{n=0}{\sum }}x\left(n\right)$
	DFT (Discrete Fourier Transform): X$\left(k\right)=\overset{N-1}{\underset{n=0}{\sum }}x\left(n\right)e^{\frac{-j2\pi nk}{N}}\text{ k}=0,\ldots ,\text{N}-1$
4. Power Spectral Sum (PSS)	PSS $=\overset{N-1}{\underset{k=0}{\sum }}\frac{1}{N}|\text{X}\left(\text{k}\right){|}^{2}$
5. Maximum Power Spectral Frequency (MPSF)	MPSF $=\arg\underset{k}{\max}\frac{1}{N}|\text{X}\left(\text{k}\right){|}^{2}$
6. Maximum Power Spectral Density (MPSD)	MPSD $=\underset{k}{\max}\frac{1}{N}|\text{X}\left(\text{k}\right){|}^{2}$
	$m_{n}=\overset{\infty }{\underset{-\infty }{\int }}\omega^{n}X\left(\omega \right)d\omega$
7. Hjorth Parameter: Activity	Activity = *m*_0_
8. Hjorth Parameter: Mobility	Mobility $={\left(\frac{m_{2}}{m_{0}}\right)}^{1/2}$
9. Hjorth Parameter: Complexity	Complexity $=\frac{{\left(\frac{m_{4}}{m_{2}}\right)}^{1/2}}{{\left(\frac{m_{2}}{m_{0}}\right)}^{1/2}}$
	Y $={\left[\xi_{i},\ldots ,\xi_{N}\right]}^{T}\;\xi_{i}=\left(x\left(t_{i}\right),x\left(t_{i}+\tau \right),\ldots ,x\left(t_{i}+\left(\text{m }-\;1\right)\tau \right)\right)$
10. Correlation Dimension (CD)	CD $=\underset{r\to 0}{\text{lim}}\frac{\log\text{C}\left(m,r\right)}{\log r}\;$ $C\left(m,r\right)=\frac{1}{m^{2}}\overset{m}{\underset{i,j=1}{\sum }}\theta \left(r-|\xi_{i}-\xi_{j}|\right)$ Where, θ is a Heaviside step function; *r* is threshold of similarity.
11. Kolmogorov Entropy (KE)	KE $=\frac{1}{\tau }\ln\frac{C\left(m,r\right)}{C\left(m+1,r\right)}$
12. Approximate Entropy (AE)	AE = Φ^*m*^(*r*)−Φ^*m*+1^(*r*) $\Phi^{m}\left(r\right)=\frac{1}{N-m+1}\overset{N-m+\;1}{\underset{i=1}{\sum }}\text{In}C_{i}^{m}\left(r\right)$ $C_{i}^{m}\left(r\right)=\frac{No.\;of\;\xi_{j}|\underset{j}{\max}|\xi_{i}-\xi_{j}|\leq r}{N-m+1}\;$ *j* ∈ [1, *N* − *m* + 1]
13. Sample Entropy (SaE)	SaE = −ln(Ψ^*m*+1^(*r*)/Ψ^*m*^(*r*)) ${\text{Ψ}}^{m}\left(r\right)=\frac{1}{N-m+1}\overset{N-m+\;1}{\underset{i=1}{\sum }}C_{i}^{m}\left(r\right)$ $C_{i}^{m}\left(r\right)=\frac{No.\;of\;\xi_{j}|\underset{j}{\max}|\xi_{i}-\xi_{j}|\leq r}{N-m}\;$ *j* ∈ [1, *N* − *m* + 1], *j* ≠ *i*
14. Lyapunov Exponent (LE)	$LE\left(i\right)=\underset{z\to \infty }{\text{lim}}\frac{1}{z}\log_{2}\frac{\left||\delta \xi_{i}\left(z\right)|\right|}{\left||\delta \xi_{i}\left(0\right)|\right|}\;$ Where, ||δξ_*i*_(*z*)|| is the distance of two neighboring points in the i-th direction at time z.
15. Singular Spectrum Entropy (SSE)	SSE $=-\;\underset{j}{\sum }p_{j}\left(\text{s}\right)\log\left(p_{j}\left(s\right)\right)$ Where, {*p*_*j*_(*s*)} is the probability distribution of singular spectrum value $\frac{s_{m}}{\underset{m}{\sum }s_{m}}$; *s*_*m*_ is the eigenvalue of Y.
16. Permutation Entropy (PE)	PE $=-\overset{m!}{\underset{i=1}{\sum }}p_{i}\left(\pi \right)\ln\left(p_{i}\left(\pi \right)\right)/\text{ln}\left(m!\right)$ Where, π is the order pattern; {*p*_*i*_(π)} is the probability distribution of π.
17. C0 Complexity	C0 $=\frac{\overset{N-1}{\underset{n=0}{\sum }}|x\left(n\right)-y\left(n\right){|}^{2}}{\overset{N-1}{\underset{n=0}{\sum }}|x\left(n\right){|}^{2}},$ $y\left(\text{n}\right)=\frac{1}{N}\overset{N-1}{\underset{k=0}{\sum }}\text{Y}\left(k\right)e^{\frac{j2k\pi n}{N}}$ $\text{Y}\left(\text{k}\right)\text{=}\left\{\begin{array}{l} \text{X(k), }{\text{|X(k)|}}^{2}\;>\text{ PSS}\\ 0,\;{\text{|X(k)|}}^{2}\;\geq \text{ PSS} \end{array}\right\}$
18. Shannon Entropy (SE)	SE $=-\underset{i}{\sum }p_{i}\left(\text{x}\left(\text{n}\right)\right)\log\left(p_{i}\left(\text{x}\left(\text{n}\right)\right)\right)\;$ Where, {*p*_*i*_(*x*(*n*))} is the probability distribution of x(n).
19. Power Spectral Entropy (PSE)	PSE $=-\underset{i}{\sum }p_{i}\left(\text{PS}\right)\log\left(p_{i}\left(\text{PSS}\right)\right)\;$ Where, {*p*_*i*_(PSS)} is the probability distribution of power spectral sum.
20. Differential Entropy (DE)	DE $=\frac{1}{2}\log\left(2\pi e\sigma^{2}\right)\;$ Where, σ is the standard deviation of x(n).

### 2.7. Recognition Algorithm

In this study, we adopted SVM to learn a binary classifier with extracted EEG features to distinguish TD group and ASD group. SVM is one of the most robust machine learning methods, being based on the statistical learning frameworks proposed by Cortes and Vapnik (1995).

Given the training data ${\left[\left\{x_{i},y_{i}\right\}\right]}_{i=1}^{n}$, SVM attempts to find the best hyperplanes with maximum margin to divide the training data into two groups according to the labels *y*_*i*_ ∈ {+1, −1}. To this end, SVM optimizes the model by minimizing a hinge loss, which can be formulated as follows:

(1) $$\begin{array}{l} \frac{1}{n}\sum_{i=1}^{n}\max\left[0,1-y_{i}\left(w^{T}x_{i}-b\right)\right]+\lambda ||w|{|}_{P} \end{array}$$

where *w* and *b* are the trainable parameters, *w* is the normal vector to the hyperplane, and *b* is the bias. The parameter λ controls the trade-off between the loss and penalty.

According to the structural risk minimization, larger margins should lead to better generalization and prevent overfitting in the high-dimensional feature spaces. The solution of SVM depends only on the points located on the two supporting planes, which are called support vectors. Hence, when compared with other machine learning methods, SVM has better generalization ability in dealing with small samples with high-dimensional features. Considering small size of the available participants with ASD and high-dimensional EEG features, SVM is more suitable for the data analysis in this study.

The second terms in Formula (1) are regularization terms with *l*_*p*_-norm penalty. When *p* = 1, *l*_1_-norm penalty forces the normal vector to satisfy sparsity. L1-norm regularization is a wrapper-based strategy for the automatic feature selection. While minimizing the structural risk of model prediction, L1-norm regularization makes unimportant features with weights of zero value being eliminated from the candidate set. The remaining features are useful for the autism screening under positive/negative emotional stimulus. Hence, it is an effective way to measure the importance and discrimination of features through the corresponding weights and eliminate the redundant and unimportant features.

## 3. Results

The discrimination of different EEG handcrafted features from different rhythms and brain regions is investigated. The selected features with *l*_1_-regularized SVM for the classification of children with ASD and TD children are evaluated. In order to make a fair comparison, the leave-one-out method is adopted to evaluate the performance of different features, i.e., each child should be used once as test data to evaluate the model trained with the data set composed of all rest children.

### 3.1. Discrimination of Different Handcrafted Features

**Figure 3:**
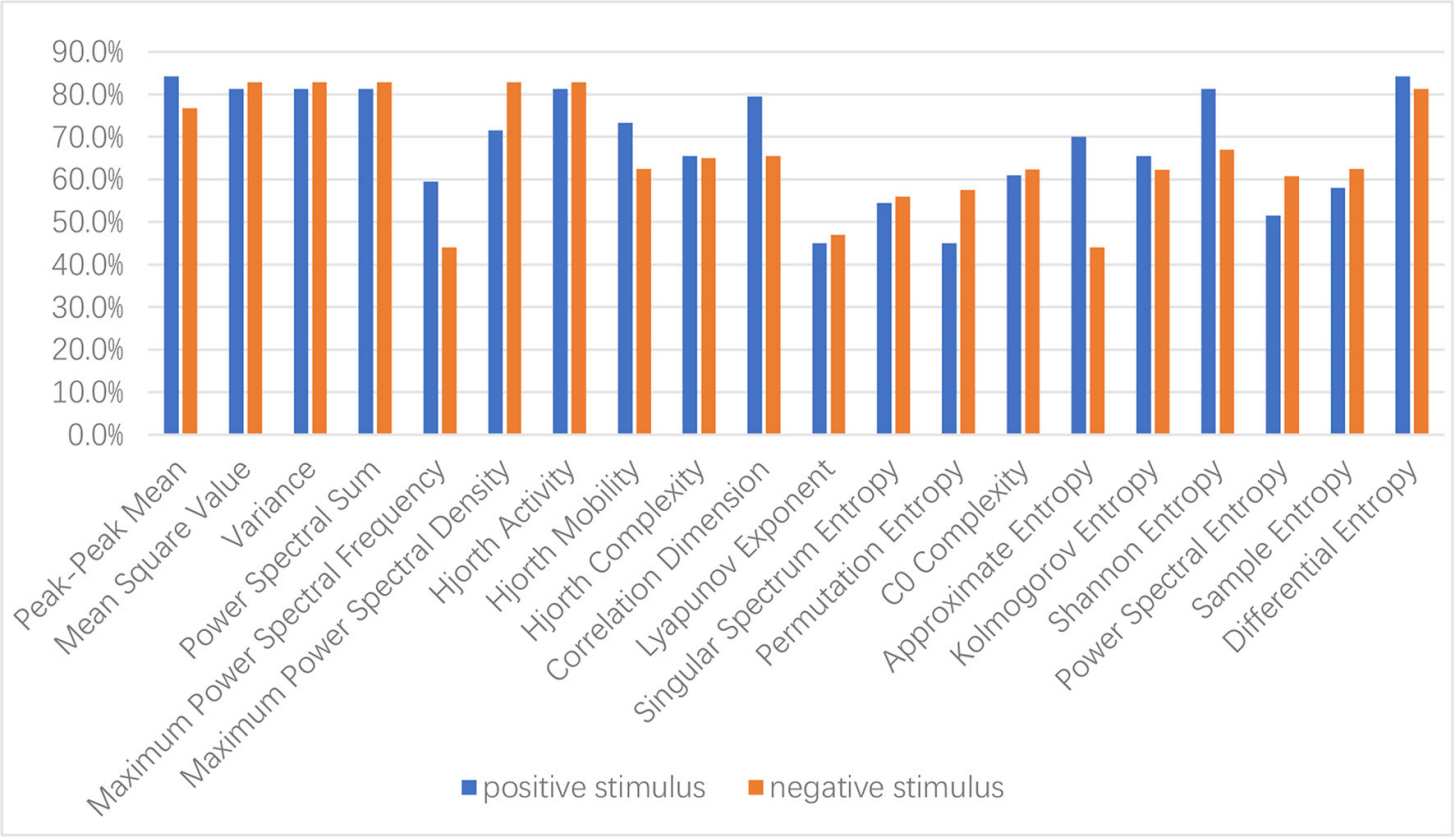
Performance comparison of different handcrafted features for typically developing (TD)/autism spectrum disorder (ASD) classification.

To explore the discrimination of different handcrafted features, each kind of handcrafted feature is extracted from five rhythms, all the electrodes are input into an SVM classifier, and the classification accuracy of a single feature is compared. The classification results are shown in Figure 3.

It is noted that under positive emotional stimulus, peak–peak mean and differential entropy have the best discrimination, both with 84.4% of classification accuracy, followed by mean square value, variance, Hjorth parameter: activity, power sum, and Shannon entropy, all with 81.3% of classification accuracy, while under negative emotional stimulus, mean square value, variance, Hjorth parameter: activity, maximum power spectral density, and power sum have the best discrimination, all with 82.8% of classification accuracy, followed by differential entropy, with 81.3% of classification accuracy. The features with above 80% of classification accuracy under two emotional stimuli include mean square value, variance, Hjorth parameter: activity, power sum, and differential entropy.

### 3.2. Discrimination of Different Rhythms

**Figure 4:**
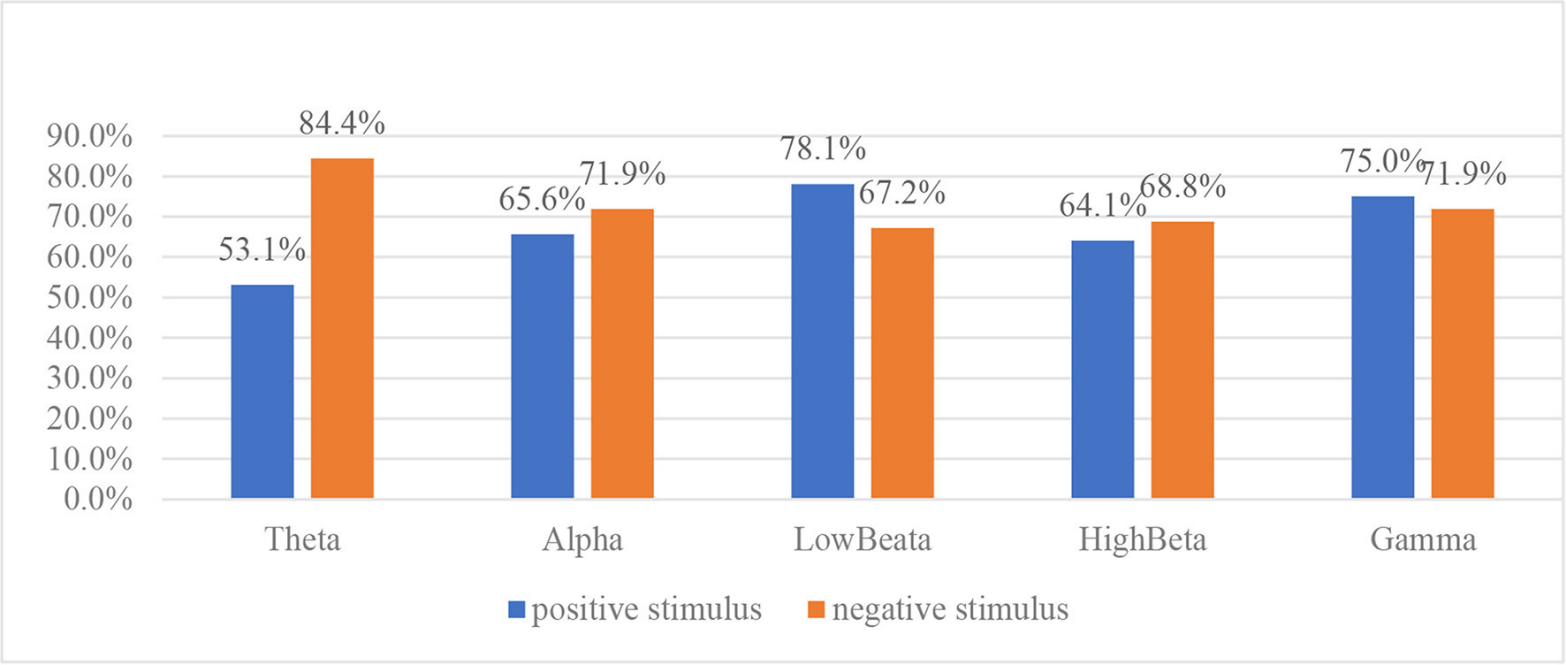
Performance comparison of different rhythm features for typically developing (TD)/autism spectrum disorder (ASD) classification.

To explore the discrimination of different rhythms, 20 kinds of handcrafted features from a single rhythm are input into an SVM classifier and the classification accuracy of features extracted from different rhythms is compared. The classification results are shown in Figure 4.

It is noted that under positive emotional stimulus, the features extracted from low beta rhythm have the best discrimination, with 78.1% of classification accuracy, followed by gamma rhythm, alpha rhythm, high beta rhythm, and theta rhythm, while under negative emotional stimulus, the features extracted from theta rhythm have the best discrimination, with 84.4% of classification accuracy, followed by alpha rhythm, gamma rhythm, high beta rhythm, and low beta rhythm. On average, the features extracted from gamma rhythm have the highest classification accuracy under two emotional stimuli.

### 3.3. Discrimination of Different Brain Regions

**Figure 5:**
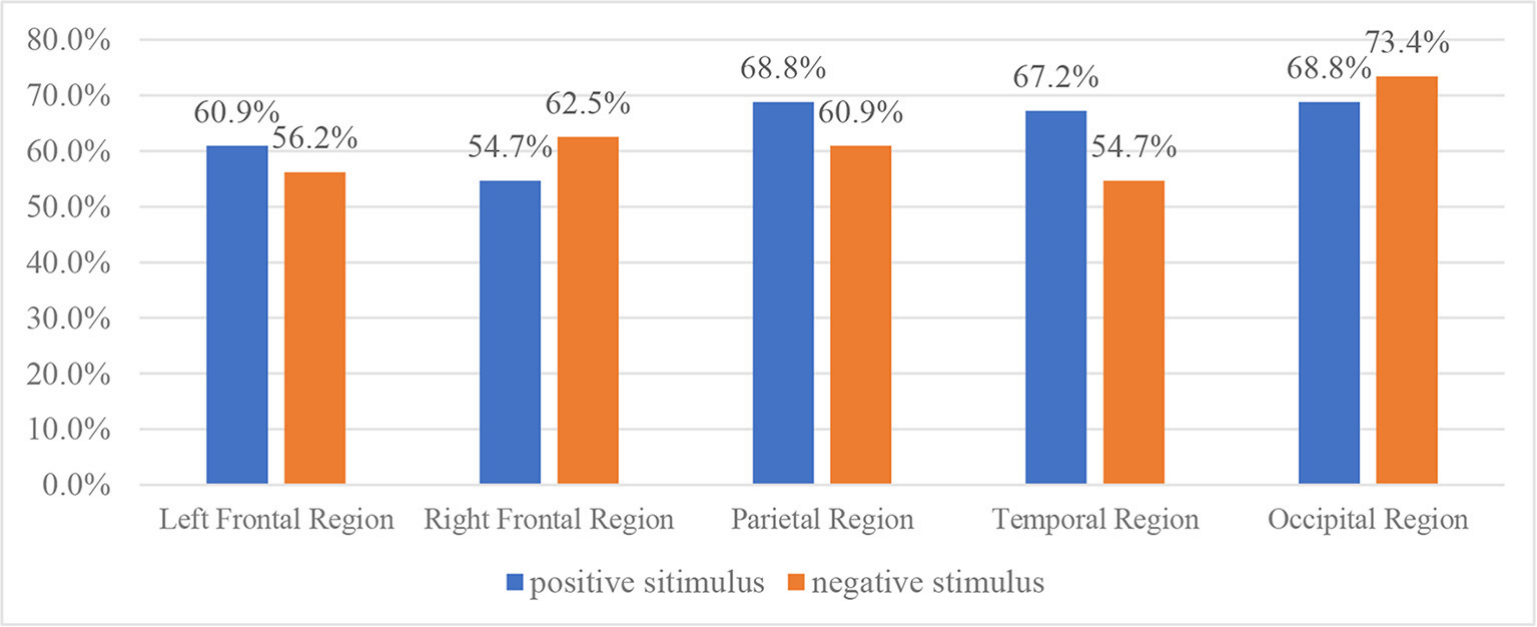
Performance comparison of different brain region features for typically developing (TD)/autism spectrum disorder (ASD) classification.

To explore the discrimination of different rhythms, 14 data acquisition electrodes are divided into 5 groups, namely, (1) the left frontal lobe (including AF3, F3, F7, and FC5), (2) the right frontal lobe (including AF4, F4, F8, and FC6), (3) the temporal lobe (including T7 and T8), (4) the parietal lobe (including P7 and P8), and (5) the occipital lobe (including O1 and O2). A total of 20 kinds of handcrafted features from electrodes of the same group are input into an SVM classifier, and the classification accuracy of features extracted from different brain regions is compared. The classification results are shown in Figure 5.

It is noted that under positive emotional stimulus, the features extracted from the temporal lobe and occipital lobe have the best discrimination, with 68.8% of classification accuracy, while under negative emotional stimulus, the features extracted from the occipital lobe have the best discrimination, with 73.4% of classification accuracy. On average, the features extracted from the occipital lobe have the highest classification accuracy under two emotional stimuli.

### 3.4. Classification With *l*_1_-Regularized SVM

To evaluate the proposed method, 20 handcrafted features are input into an ordinary linear SVM and an *l*_1_-regularized SVM, respectively, which adaptively select good features to distinguish ASD group and TD group. The λ is set between 0.1 and 1, and step length is set to 0.01 to investigate the effect of parameter λ on the classification accuracy. The results are shown in Table 2.

**Table 2:** The highest classification accuracy using the ordinary linear support vector machine (SVM) and the *l*_1_-regularized SVM, respectively.

**Methods**	**Stimulus**	**λ**	**No. of selected features**	**Accuracy (%)**
Ordinal linear SVM	Positive	–	–	81.3
	Negative	–	–	78.1
*l*_1_-regurarized SVM	Positive	0.13	10	93.8
	Negative	0.24	21	87.5

When compared with the ordinal linear SVM classification, the highest screening accuracies of children with ASD under positive and negative emotional stimuli are 81.3 and 78.1%, respectively, where the proposed method achieved the highest recognition accuracy of 93.8% when λ = 0.13 under positive emotional stimulus, and 87.5% when λ = 0.24 under negative emotional stimulus.

When λ = 0.13, 10 features with non-zero weights were selected for ASD/TD classification under positive emotional stimulus, which are listed in Table 3. It is noted that eight kinds of handcrafted features were selected, of which correlation dimension and maximum power spectral frequency were selected twice. In terms of rhythms, five features from gamma rhythm were selected, which showed that gamma rhythm had better discrimination under positive emotional stimulus. In terms of the brain region, three features from the right frontal lobe were selected, which showed that the right frontal lobe had better discrimination under positive emotional stimulus.

**Table 3:** The features selected by the *l*_1_-regularized support vector machine under positive emotional stimulus.

**No**.	**Features**	**Rhythm**	**Brain region**
1	Differential entropy	Alpha	Left frontal lobe
2	C0 complexity	Theta	Left frontal lobe
3	Correlation dimension	Gamma	Temporal lobe
4	Hjorth parameter: mobility	High Beta	Parietal lobe
5	Maximum power spectral frequency	Low Beta	Occipital lobe
6	Correlation dimension	Gamma	Parietal lobe
7	Singular spectrum entropy	Alpha	Temporal lobe
8	Maximum power spectral frequency	Gamma	Right frontal lobe
9	Variance	Gamma	Right frontal lobe
10	Power spectral entropy	Gamma	Right frontal lobe

When λ = 0.24, 21 features with non-zero weights were selected for ASD/TD classification under negative emotional stimulus, which are listed in Table 4. It is noted that 14 kinds of handcrafted features were selected, of which correlation dimension was selected 4 times. In terms of rhythms, seven features from gamma rhythm were selected, which showed that gamma rhythm also had better discrimination under negative emotional stimulus. In terms of brain region, eight features from the occipital lobe were selected, which showed that the occipital lobe had better discrimination under negative emotional stimulus.

**Table 4:** The features selected by the *l*_1_-regularized support vector machine under negative emotional stimulus.

**No**.	**Features**	**Rhythm**	**Brain region**
1	Approximate entropy	High Beta	Left frontal lobe
2	Power spectral entropy	Theta	Left frontal lobe
3	Approximate entropy	Low Beta	Left frontal lobe
4	Sample entropy	Alpha	Temporal lobe
5	Lyapunov exponent	High Beta	Occipital lobe
6	Correlation dimension	Alpha	Occipital lobe
7	Peak–peak mean	Gamma	Occipital lobe
8	Mean square value	Gamma	Occipital lobe
9	Variance	Gamma	Occipital lobe
10	Maximum power spectral density	Gamma	Occipital lobe
11	Hjorth parameter: mobility	Alpha	Occipital lobe
12	Correlation dimension	Gamma	Occipital lobe
13	Hjorth parameter: mobility	Theta	Parietal lobe
14	Correlation dimension	Gamma	Parietal lobe
15	Correlation dimension	Gamma	Temporal lobe
16	Kolmogorov entropy	High Beta	Right frontal lobe
17	Power sum	Alpha	Right frontal lobe
18	Maximum power spectral frequency	Low Beta	Right frontal lobe
19	Sample entropy	High Beta	Right frontal lobe
20	Hjorth parameter: activity	High Beta	Right frontal lobe
21	Sample entropy	High Beta	Right frontal lobe

## 4. Conclusion

In order to **a**im for screening children with ASD, this study proposes a linear SVM based on L1-norm regularization, which readily handles the classification of children with autism, with the selected EEG features collected under positive and negative emotional stimuli. To evaluate the performance of the proposed algorithm, we designed an ERP experimental paradigm. We stimulated the subjects with positive and negative emotions, and in the meantime, the evoked EEG data sets were collected. We also linked the EEG signal and social and emotional behaviors to further analyze the pathogeny of children with ASD. Then, the EEG features were obtained for the screening of autism. When compared with the existing work that studied a limited number of features and lacked adequately strong representation ability, this study extracts a wider range of 20 types of the EEG features for the screening of autism. However, increasing the number of features resulted in the redundancy and complementarity of multiple features; to solve this problem, we proposed a linear SVM based on the L1-norm feature selection algorithm to improve the accuracy of screening for children with ASD. The proposed algorithm displayed significant advantages over the existing benchmark, i.e., in the real data set tests, compared with the ordinal linear SVM classification, the accuracies of screening children with autism under positive and negative emotional stimuli were dramatically increased from 81.3 and 78.1% to 93.8 and 87.5%. Our experiments proved that the L1-norm can remove redundant features, screen out better features, and improve the accuracy of screening. In addition, the experimental results suggested that differential entropy of the EEG collected from positive and negative emotional stimuli was able to screen autism, and the differences in emotion could be identified using this feature. We also found that the highest averaged classification accuracy appeared in the occipital lobe under both positive and negative emotional stimuli, which could be the notification point of the difference between TD children and children with ASD.

## Data Availability Statement

The raw data supporting the conclusions of this article will be made available by the authors, without undue reservation.

## Ethics Statement

The studies involving human participants were reviewed and approved by Ethics Committee of Central China Normal University. Written informed consent to participate in this study was provided by the participants' legal guardian/next of kin.

## Author Contributions

SP proposed the idea, conducted the experiments, and wrote the manuscript. RX, XY, and XH provided advice on the research approaches, guided the experiments, and checked and revised the manuscript. LiL and LeL offered important help on EEG processing and analysis methods. All authors contributed to the article and approved the submitted version.

## Conflict of Interest

The authors declare that the research was conducted in the absence of any commercial or financial relationships that could be construed as a potential conflict of interest.

## References

[B1] Acharya U. R. Fujita H. Sudarshan V. K. Bhat S. Koh J. E. W. (2015). Application of entropies for automated diagnosis of epilepsy using EEG signals: a review. Knowl. Based Syst. 88, 85–96. 10.1016/j.knosys.2015.08.004

[B2] American Psychiatric Association (2013). Diagnostic and Statistical Manual of Mental Disorders, 5th Edn. Washington, DC: American Psychiatric Association.

[B3] Badcock N. A. Mousikou P. Mahajan Y. de Lissa P. Thie J. McArthur G. (2013). Validation of the Emotiv EPOC EEG gaming system for measuring research quality auditory ERPs. PeerJ 1:e38. 10.7717/peerj.38 23638374 PMC3628843

[B4] Badcock N. A. Preece K. A. de Wit B. Glenn K. Fiederr N. Thie J.. (2015). Validation of the Emotiv EPOC EEG system for research quality auditory event-related potentials in children. PeerJ 3:e907. 10.7717/peerj.907 25922794 PMC4411518

[B5] Barton M. L. Dumont-Mathieu T. Fein D. (2012). Screening young children for autism spectrum disorders in primary practice. J. Autism Dev. Disord. 42, 1165–1174. 10.1007/s10803-011-1343-5 21842325

[B6] Bickel J. Bridgemohan C. Sideridis G. Huntington N. (2015). Child and family characteristics associated with age of diagnosis of an autism spectrum disorder in a tertiary care setting. J. Dev. Behav. Pediatr. 36, 1–7. 10.1097/DBP.0000000000000117 25539088

[B7] Castro-Cabrera P. Gomez-Garcia J. Restrepo F. Moscoso O. Castellanos-Dominguez G. (2010). Evaluation of feature extraction techniques on event-related potentials for detection of attention-deficit/hyperactivity disorder, in Proceedings of 2010 Annual International Conference of the IEEE Engineering in Medicine and Biology Society (New York, NY: IEEE Press), 851–854. 10.1109/IEMBS.2010.5626862 21096317

[B8] Chen D. Tang Y. Zhang H. Wang L. Li X. (2021). Incremental factorization of big time series data with blind factor approximation, in IEEE Transactions on Knowledge and Data Engineering, Vol. 33 (IEEE), 569–584 10.1109/TKDE.2019.2931687

[B9] Cortes C. Vapnik V. (1995). Support-vector networks. Mach. Learn. 20, 273–297. 10.1007/BF00994018

[B10] Delgado-Bonal A. Marshak A. (2019). Approximate entropy and sample entropy: a comprehensive tutorial. Entropy 21:541. 10.3390/e21060541 33267255 PMC7515030

[B11] Dong H. Chen D. Zhang L. Ke H. Li X. (2021). Subject sensitive EEG discrimination with fast reconstructable CNN driven by reinforcement learning: A case study of ASD evaluation. Neurocomputing 449, 136–145. 10.1016/j.neucom.2021.04.009

[B12] Duan R. N. Zhu J. Y. Lu B. L. (2013). Differential entropy feature for EEG-based emotion classification, in 6th International IEEE/EMBS Conference on Neural Engineering (NER) (New York, NY: IEEE Press), 81–84. 10.1109/NER.2013.6695876

[B13] Duvinage M. Castermans T. Petieau M. Hoellinger T. Cheron G Dutoit T. (2013). Performance of the emotiv epoc headset for P300-based applications. Biomed. Eng. Online 12:56. 10.1186/1475-925X-12-56 23800158 PMC3710229

[B14] Esguerra J. P. H. Keh O. S. C. T. Chupungco A. M. A. (2012). Nonlinear time series analysis of electroencephalogram tracings of children with autism. Int. J. Bifurcat. Chaos 22:1250044. 10.1142/S0218127412500447

[B15] Fan J. Wade J. W. Key A. P. Warren Z. E. Sarkar N. (2017). EEG-based affect and workload recognition in a virtual driving environment for ASD intervention. IEEE Transac. Biomed. Eng. 65, 43–51. 10.1109/TBME.2017.2693157 28422647 PMC5638702

[B16] Gross J. J. Levenson R. W. (1995). Emotion elicitation using films. Cogn. Emot. 9, 87–108. 10.1080/02699939508408966

[B17] Guyon I. Elisseeff A. (2003). An introduction to variable and feature selection. J. Mach. Learn. Res. 3, 1157–1182.

[B18] Hjorth B. (1970). EEG analysis based on time domain properties. Electroencephalogr. Clin. Neurophysiol. 29, 306–310. 10.1016/0013-4694(70)90143-4 4195653

[B19] Jung T. P. Humphries C. Lee T. W. Makeig S. Mckeown M. J. Iragui V.. (1998). Removing electroencephalographic artifacts: comparison between ICA and PCA, in Neural Networks for Signal Processing VIII. Proceedings of the 1998 IEEE Signal Processing Society Workshop (Cat. No.98TH8378) (Cambridge: IEEE).

[B20] Kiyoto K. Yuki K. Hitoshi K. Hidenori Y. (2007). Neuroimaging in autism spectrum disorders. Neurosci. Res. 58:S27. 10.1016/j.neures.2007.06.158

[B21] Lei M. Meng G. Zhang W. M. Sarkar N. (2016). Sample entropy of electroencephalogram for children with autism based on virtual driving game. Acta Phys. Sin. 65:108701. 10.7498/aps.65.108701

[B22] Li X. Song D. Zhang P. Zhang Y. Hou Y. Hu B. (2018). Exploring EEG features in cross-subject emotion recognition. Front. Neurosci. 12:162. 10.3389/fnins.2018.00162 29615853 PMC5867345

[B23] Lobar S. L. (2016). DSM-V Changes for Autism Spectrum Disorder (ASD): implications for diagnosis, management, and care coordination for children with ASDs. J. Pediatr. Health Care 30, 359–365. 10.1016/j.pedhc.2015.09.005 26602109

[B24] Polyak A. Kubina R. M. Girirajan S. (2015). Comorbidity of intellectual disability confounds ascertainment of autism: implications for genetic diagnosis. Am. J. Med. Genet. Part B Neuropsychiatr. Genet. 168, 600–608. 10.1002/ajmg.b.32338 26198689

[B25] Rottenberg J. Gross J. J. Wilhelm F. H. Najmi S. Gotlib I. H. (2002). Crying threshold and intensity in major depressive disorder. J. Abnorm. Psychol. 111, 302–312. 10.1037/0021-843X.111.2.302 12003451

[B26] Schuster H. G. (1995). Deterministic Chaos: An Introduction, 3rd Edn. New York, NY: Wiley.

[B27] Shams W. K. Wahab A. (2013). Source-temporal-features for detection EEG behavior of autism spectrum disorder, in Proceedings of 5th International Conference on Information and Communication Technology for the Muslim World (New York, NY: IEEE Press), 1–5.

[B28] Tierney A. L. Gabard-Durnam L. Vogel-Farley V. Tager-Flusberg H. Nelson C. A. (2012). Developmental trajectories of resting EEG Power: an endophenotype of autism spectrum disorder. PLoS ONE 7:e39127. 10.1371/journal.pone.0039127 22745707 PMC3380047

[B29] Zhao J. Ding M. Tong Z. Han J. Li X. Kang J. (2019). Feature exaction and classification of autism spectrum disorder children related electroencephalographic signals based on entropy. J. Biomed. Eng. 36, 183–188. 10.7507/1001-5515.201709047 31016933 PMC9929912

